# Chondrocyte outgrowth into a gelatin scaffold in a single impact load model of damage/repair – effect of BMP-2

**DOI:** 10.1186/1471-2474-8-120

**Published:** 2007-12-05

**Authors:** Frances MD Henson, Thea Vincent

**Affiliations:** 1 Department of Veterinary Medicine, University of Cambridge, Madingley Road, Cambridge CB3 0ES, UK

## Abstract

**Background:**

Articular cartilage has little capacity for repair *in vivo*, however, a small number of studies have shown that, *in vitro*, a damage/repair response can be induced. Recent work by our group has shown that cartilage can respond to single impact load and culture by producing repair cells on the articular surface. The purpose of this study was to identify whether chondrocyte outgrowth into a 3D scaffold could be observed following single impact load and culture. The effect of bone morphogenic-2 (BMP-2) on this process was investigated.

**Methods:**

Cartilage explants were single impact loaded, placed within a scaffold and cultured for up to 20 days +/- BMP-2. Cell numbers in the scaffold, on and extruding from the articular surface were quantified and the immunohistochemistry used to identify the cellular phenotype.

**Results:**

Following single impact load and culture, chondrocytes were observed in a 3D gelatin scaffold under all culture conditions. Chondrocytes were also observed on the articular surface of the cartilage and extruding out of the parent cartilage and on to the cartilage surface. BMP-2 was demonstrated to quantitatively inhibit these events.

**Conclusion:**

These studies demonstrate that articular chondrocytes can be stimulated to migrate out of parent cartilage following single impact load and culture. The addition of BMP-2 to the culture medium quantitatively reduced the repair response. It may be that the inhibitory effect of BMP-2 in this experimental model provides a clue to the apparent inability of articular cartilage to heal itself following damage *in vivo*.

## Background

Articular cartilage constantly experiences the damaging effects of biomechanical 'wear and tear' and is reported to have little capacity for permanent repair due primarily to its limited proliferative capability [1]. This limited repair capacity leads to damage accumulation, resulting in the loss of cartilage integrity and the development of chronic degenerative joint disease, specifically osteoarthritis (OA) [2].

Cartilage repair is an important clinical challenge and is being studied extensively.

Work undertaken by our group has recently described a novel intrinsic damage repair response in mature equine articular cartilage explants following single impact load and subsequent culture [3]. This response is characterised by the appearance of chondrocyte repair cells on damaged cartilage. This apparent mobilisation of chondrocytes has also been described following significant cartilage damage (mincing), where the chondrocytes were observed to be migrating into a 3D scaffold. This scaffold was subsequently used to repair cartilage lesions *in vivo *[4]. This work demonstrates that cartilage can be stimulated sufficiently to mobilise chondrocytes to sites of damage and beyond, into a scaffold, and that these mobilised cells have distinct and exciting clinical possibilities.

In the damage/repair model described by our group [3] we have shown that repair cell numbers are significantly increased when the cartilage is cultured in the presence of 50 ng/ml fibroblastic growth factor-2 (FGF-2). The mechanism by which repair cells appear on the surface of damaged cartilage is unknown, however we have previously hypothesised that FGF-2 (both exogenous and from endogenous release [5,6]) is driving this process. FGF-2 is one of a family of growth factors that have profound effects on cartilage development and repair. Other growth factors are also clearly involved in cartilage biology and of these the bone morphogenic proteins (BMPs) are some of the most widely studied. In particular it has been shown that BMP-2 promotes chondrogenesis [7], induces the differentiation of stem cells into chondrocytes [8] is shown to be up-regulated in cartilage after mechanical damage [9] and promotes articular cartilage repair experimentally [10]. BMP-2 is therefore a growth factor of potential interest in *in vitro *cartilage repair.

The aims of this study were (i) to identify whether repair cells produced following SIL and subsequent culture have the ability to migrate out of parent cartilage into a 3D gelatin scaffold and ii) to investigate the effects of 100 ng/ml BMP-2 on these events.

## Methods

### Horses

Cartilage was obtained from horses aged between 7 and 9 years (n = 3) that were humanely destroyed for reasons other than joint disease. For this study, only normal, healthy cartilage was used based on the lack of pathology following macroscopical and microscopical examination.

### Harvesting of cartilage

Cartilage discs (7 mm diam.) were dissected aseptically from the articular surface of the proximal phalanx, without attached subchondral bone, using a sterile cork borer. Discs were placed into sterile phosphate-buffered saline (PBS) containing antibiotics and antimycotics (200 IU/ml penicillin, 2.5 μg/ml fungizone, 100 μg/ml streptomycin, 20 μg/ml gentamycin, Invitrogen, Paisley, Scotland), and then washed a further 3 times in sterile PBS.

### Impact loading of cartilage discs

Discs were randomly divided into two groups – control (unimpacted) or impacted. Discs were impacted using a drop tower device following the method described previously [3,11,12]. Each disc, with the articular surface facing down was impacted from a height of 2.5 cm using a weight of 500 g. The approximate impact energy applied to each disc was 0.175 J, impacted at a velocity of approximately 0.7 m/s. To ensure constant compression conditions, the impactor was left upon the disc for 10 s before being removed.

### In vitro culture of cartilage discs

After impact, discs were placed into pockets created by sharp excision in a gelatin scaffold (Gelfoam, Pharmacia Upjohn, USA) (1 cm × 1 cm pieces). Cartilage+Gelfoam units were cultured for 0, 10 and 20 days in Dulbecco's modified Eagle's medium (DMEM, Sigma-Aldrich, UK) supplemented with 200 IU/ml penicillin (Invitrogen, UK), 2.5 υg/ml streptomycin (Invitrogen, UK), 500 υg/ml ascorbic acid (Sigma Aldrich, UK) and 10% fetal calf serum (Invitrogen, UK) at 37 degrees C and 5% CO2.) Groups of discs were also cultured in the presence of 100 ng/ml BMP-2 (Sigma Aldrich, UK). 100 ng/ml BMP-2 was used in this experiment as 100 ng/ml has been shown to be an effective dose for stimulation of chondrocyte and mesenchymal stem cell responses [13,14]. Each experimental time point was made up of 3 cartilage discs i.e each experiment was performed in triplicate in each of the three animals. Control explant discs (not impacted) were cultured for the same time-periods. At the end of each time point cartilage discs were embedded in Tissue Tek OCT (Sakura Finetek Europe, The Netherlands) and snap-frozen in liquid nitrogen or placed immediately into formal saline and paraffin embedded. Frozen sections (10 μm) were cut and placed on poly-L-lysine coated slides for histological and immunocytochemical analysis.

### Histological and immunocytochemical analysis

Sections were stained for routine histological analysis with Haematoxylin and Eosin (Sigma Aldrich). The following features of each experimental time point were quantified cells with elongated shape, cells with pyknotic nuclei, cells on the surface of the section, cells extruded from the section and cells trapped in gelfoam. The quantification of each of these cell features was performed by counting 3 adjacent high power fields in the top, middle and bottom of the cartilage section using a standardised grid approach.

Identification of the synthesis of hyaline cartilage specific proteins was performed by standard immunolocalisation using the following primary antibodies – polyclonal rabbit anti-rat collagen IX/XI (Calbiochem/Merck), polyclonal rabbit anti-porcine collagen type II (raised and characterised by M. E. Davies), polyclonal mouse anti-bovine collagen type I(Sigma-Aldrich) and polyclonal goat anti-rabbit fibronectin (Sigma, UK). These antibodies have been previously shown to recognise equine epitopes ([15,16]). The appropriate FITC-conjugated secondary antibodies were used to visualise the antigen.

### Quantitation techniques

Data from all sections was pooled. Chondrocyte numbers were expressed as the percentage of cells as a total of the number of cells within the section. This calculated figure was used in order to minimise the effect of different cells numbers within sections from different animals. Statistical significance was identified suing Mann-Whitney and Kruskal-Wallis tests.

## Results

### Cartilage damage following a single impact load

Cartilage discs that had been impacted suffered damage at the articular surface immediately upon impact. This damage was characterised by loss of proteoglycan (as demonstrated by loss of metachromatic staining), roughening of the articular surface and fissure formation, as previously described ([17,18]). All control explants, in contrast, presented an intact articular surface and uniform metachromatic staining, with no apparent loss of proteoglycan.

### Quantification of chondrocytes in scaffold

**Figure 1:**
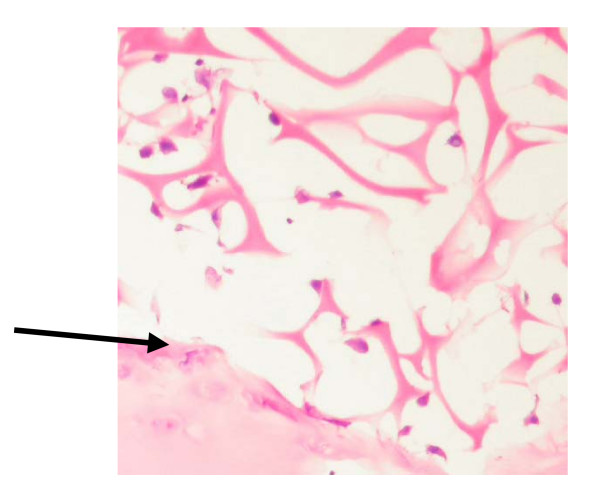
Histological section showing the junction between the articular surface and the gelfoam scaffold. Cells have migrated out of the cartilage (bottom left of picture) and are clearly seen associated with the gelatin 'fibres' The arrow marks the Gelfoam-cartilage junction. Stained with H&E. ×200.

At t = 0 all chondrocytes were observed to be within the parent bone as this time point represents the intial state of the cartilage prior to culture. When cultured, chondrocytes were observed within the gelfoam scaffold at day 11 and day 20 under all experimental conditions, including non impacted cartilage. Chondrocytes were usually observed to be closely associated with gelfoam 'fibres' suggesting that they may be adherent to the scaffold (Figure 1).

**Figure 2:**
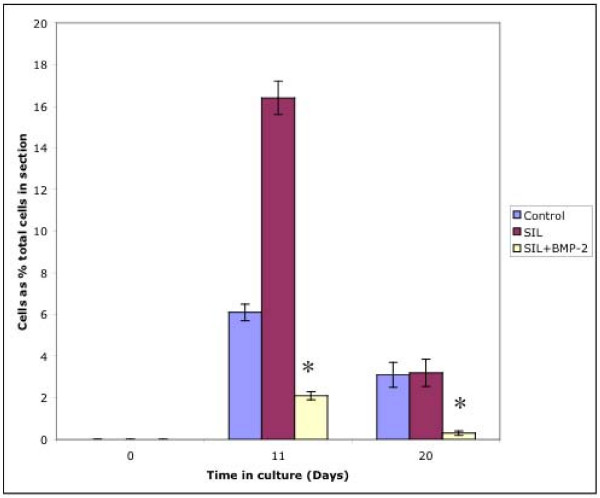
Graph to show numbers of cells captured in 3D gelatin scaffold ('Gelfoam'). It can be seen that cells were detected in the Gelfoam scaffold at days 11 and 20 in all three culture conditions. At day 11 and day 20 there were significantly reduced numbers of chondrocytes in the Gelfoam in the samples cultured in the presence of 100 ng/ml BMP-2 (*). At day 20 there was a significant decrease in cell numbers in all three experimental conditions.

The chondrocytes observed within the gelfoam at d11 and d 20 were quantified as a percentage of cells within the parent section. There was no significant difference between the numbers of chondrocytes in the gelfoam between control and SIL sections at d11 or d20, however there was a statistically significant difference decrease in the samples cultured with BMP-2 (p = 0.01 at day 11 compared to SIL sections, p = 0.049 compared to control sections, p = 0.03 at day 20 compared to SIL sections, p = 0.03 compared to control sections). When the number of chondrocytes within the gelfoam at days 11 and 20 was compared, it was shown that the percentage of chondrocytes in the gelfoam had significantly decreased between from d11 to d20 in control (P = 0.005) and SIL (P = 0.007) sections (Figure 2). There was no significant change in the number of cells that had entered the gelfoam scaffold from the sections supplemented with BMP-2 over time.

### Cells extruded from sections

**Figure 3:**
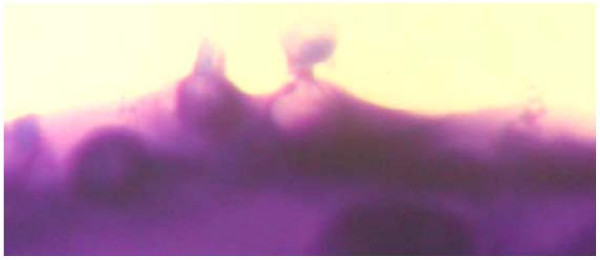
Histological section of the articular surface edge following SIL and culture for 20 days showing two adjacent chondrocytes extruding out of the parent articular cartilage. There appears to be a breach in the surface of the cartilage and cytoplasm appears to be moving out of the cartilage. Stained with toluidine blue. ×600.

**Figure 4:**
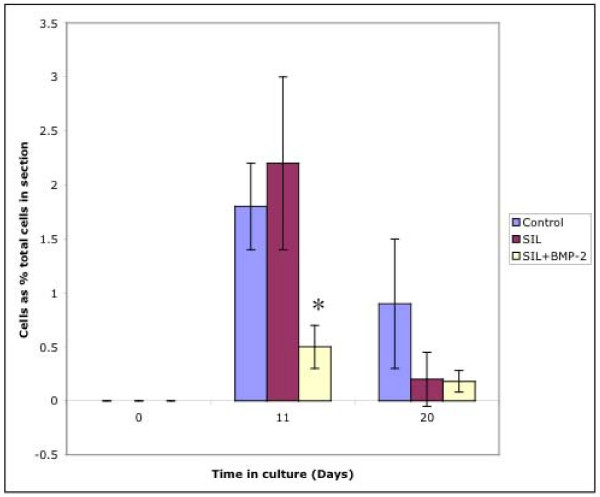
Graph to show numbers of cells extruding out of the articular cartilage surface. It can be seen that cells were observed to be extruding at days 11 and 20 in all three culture conditions. At day 11 there was a significant decrease in the number of cells extruding from the cartilage surface in the samples cultured in the presence of 100 ng/ml BMP-2 (*) compared to both control and SIL sections.

Cells were considered to be extruded from the parent cartilage if they could be observed actually exiting out of the articular surface (Figure 3). In all cases there appeared to be a breach in the articular surface through which cytoplasm was apparently moving. Chondrocytes were observed to be extruding from the parent cartilage at days 11 and 20 under all culture conditions studied (Figure 4). At t = 0 no chondrocytes were observed to be extruding i.e. extrusion of cells from the cartilage did not appear to occur prior to culturing. There was no significant difference in the number of cells extruding from the parent cartilage in control and SIL cartilage, however, in the SIL+BMP sections there was a statistically significant decrease in the number of cells extruding at day 11 compared to controls (p = 0.04) and SIL sections (p = 0.05) (Figure 4).

### Cells on the articular surface

**Figure 5:**
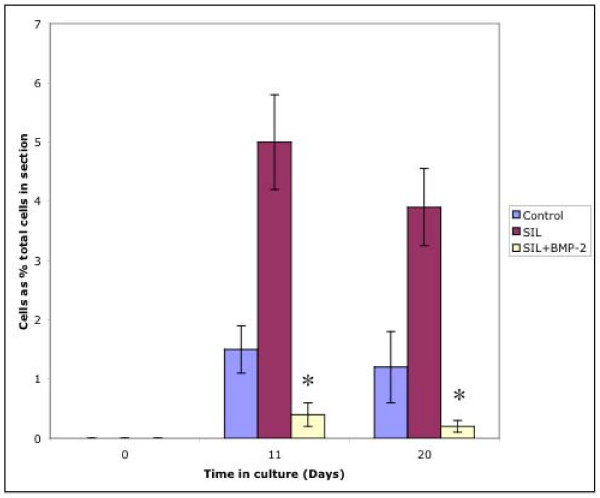
Graph to show numbers of cells observed on the articular cartilage surface. It can be seen that cells were observed on the articular surface at days 11 and 20 in all three culture conditions. At day 11 and day 20 there was a significant decrease in the number of cells on the cartilage surface in the samples cultured in the presence of 100 ng/ml BMP-2 (*) compared to both control and SIL sections.

No cells were detected on the articular surface at day 0. Cells were detected on the articular surface at day 11 and day 20 under all experimental conditions (Figure 5). There was no significant difference in the number of cells on the articular surface between control and SIL cartilage at either day 11 or 20, however the number of cells on the articular surface of the SIL+BMP-2 sections were statistically decreased compared to both control and SIL, (p = 0.03 at day 11 compared to SIL sections, p = 0.02 compared to control sections, p = 0.02 at day 20 compared to SIL sections, p = 0.015 compared to control sections).

When the number of cells on the articular surface was analysed over time, there was no statistical difference between d11 and d20 in control or SIL sections. The number of cells at the articular surface in SIL+BMP-2 decreased significantly between d11 and d20 (P = 0.05)

### Shape changes

**Figure 6:**
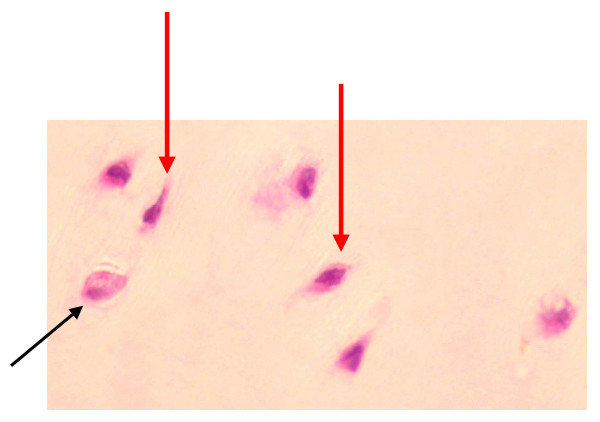
Histological section of cartilage after SIL and culture in the presence of BMP-2 for 20 days. A normal rounded chondrocytes is seen (black arrow) in the same field as elongated chondrocytes (red arrows). Stained with H&E. ×300.

**Figure 7:**
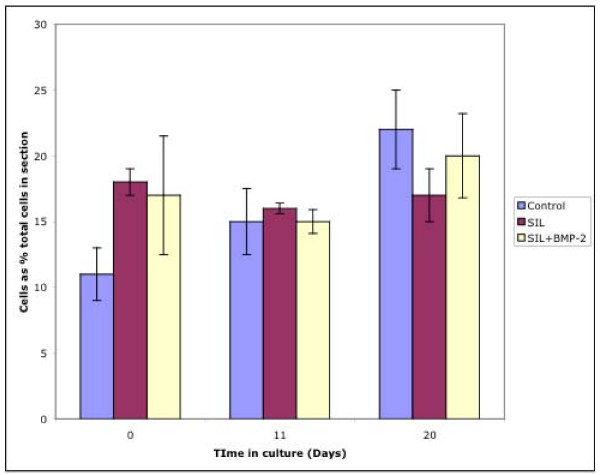
Graph to show the percentage of cells considered as having shape changes within the cartilage. It can be seen that cells with an elongated phenotype were present at all time points in all three culture conditions. There was no statistically significant difference in cell shape between culture conditions.

A chondrocyte was considered to have undergone a shape change when there was a clear change from the usual rounded phenotype to an elongated phenotype (Figure 6). During the culture period there was a relatively high level of shape change under all experimental conditions at all time points (Figure 7). At time = 0 there was an approximately 10% level of elongated cells within the cartilage in control cartilage. This was apparently increased following SIL, although this was not significant. Following culture there was no change in the numbers of elongated cells at day 11 or 20 between different treatment groups.

### Pyknotic cell death

There was no evidence of pyknotic cell death in the chondrocytes within the gelfoam, on the articular surface or extruding from the cartilage.

### Phenotypic analysis of captured cells

Immunohistochemical techniques revealed that the captured cells were type I collagen negative, type II collagen and fibronectin positive and that they stained positively for proteoglycans as assessed by toluidine blue staining.

## Discussion

This study demonstrates that, following dissection damage and/or SIL and culture, chondrocyte repair cells extrude out from the parent cartilage and can migrate into a 3D gelatin scaffold. This sequence of events is quantitatively reduced in the presence of 100 ng/ml BMP-2.

In normal cartilage, at time 0, there is no evidence of any chondrocytes outside the extracellular matrix (ECM). However, outgrowth of cells into the gelatin scaffold was observed under all experimental conditions i.e. control (no impact), SIL and SIL+BMP.

Observation of the gelfoam at days 11 and 20 of culture revealed the presence of cells adjacent to the scaffold. In order to identify the phenotype of these cells it was necessary to investigate the production of ECM macromolecules by these cells in the scaffold. This was done primarily to ascertain whether the the cells were producing hyaline cartilage specific proteins i.e. to prove that they were chondrocytes. This was achieved by immunofluorescence techniques, using antisera that we have previously demonstrated to cross react in equine cartilage. The immunofluorescence revealed that these cells were positively stained for collagen type II and fibronectin and did not stain for type 1 collagen. This indicated that the cells in the scaffold were chondrocytes and that they had no de-differentiated into fibroblasts – an important distinction to make. This observation is in agreement with other authors who have demonstrated that ability of chondrocytes to retain their phenotype in 3D scaffolds [19,20]. The ability to maintain phenotype has important implications for the use of chondrocytes captured into a scaffold in the repair of lesions as they are continuing to secrete a hyaline cartilage extracellular matrix which is vital for the re-formation of the articular cartilage surface.

As stated above, chondrocyte outgrowth into the scaffold was observed in control, SIL and SIL+BMP-2 sections. Therefore it can be concluded that the cartilage was stimulated sufficiently, in all of these experiments, to induce chondrocytes out into a gelfoam scaffold. Whilst there were moderately large numbers of cells in the gelfoam scaffold at day 11, the number of chondrocytes in the scaffold fell at day 20 indicating the cells either migrate through the gelfoam and are lost into the culture medium or that they are dying on the scaffold. However, there was no evidence of pyknotic cell death detected histologically among the chondrocytes in the scaffold and so migration out appears the most likely cause of this cell loss.

In addition to observing trends over the time course of the experiment, the numbers of chondrocytes detected on the articular surface was also quantified in order to identify any differences between experimental conditions. Whilst there were no significant differences between control and SIL sections, at both day 11 and day 20 there was a statistically significant decrease in the number of cells in the scaffold in SIL+BMP-2 sections.

The exact details of the process by which the chondrocytes migrate into the scaffold is not known. However, from basic principles, it is clear that they must migrate out of the parent cartilage in order to gain access to the scaffold. In this study we observed no evidence of cells extruding from the cartilage at d = 0, confirming that the chondrocytes are usually confined to the ECM, as is, of course, well known. Following a period in culture we observed chondrocytes 'extruding' out of the articular surface i.e. caught in the process of squeezing out of the matrix and sitting adjacent to the articular surface. In order to investigate the effect of time in culture and the effect of different culture conditions on the numbers of chondrocytes extruding from the matrix the numbers of cells extruding was quantified. In control and SIL sections there was a statistically significant decrease in the number of cells extruding from the cartilage at day 20 compared to day 11, but no difference in the number of cells adjacent to the articular surface. However, there was no difference between control sections and those sections that had been SIL either in the numbers of cells on the articular surface or extruding from the cartilage. Interestingly, sections that were SIL+BMP-2 had significantly less cells extruding from the matrix and sitting adjacent to the articular surface compared to the other experimental conditions at both day 11 and day 20.

The mechanisms driving this migration out of the parent cartilage may be due to endogenous FGF-2 release as previously discussed [3,5,6]. FGF-2 has long been associated with cell motility [21,22] and could well be playing a role in stimulating chondrocyte movement in response to damage in this experimental system; interestingly FGF-2 has been demonstrated to induce motility in previously quiescent/non motile endothelial cells *in vitro *[23] and could, thus, potentially stimulate previously quiescent chondrocytes. In the experiments described in this study there was no appreciable difference in the numbers of cell in the gelfoam scaffold or on the cartilage surface or extruding from the cartilage between control sections and SIL sections. Thus the trigger factor for this chondrocyte behaviour must lie outside the SIL damage in this experimental design. It has been shown that damage to cartilage can occur in many ways, including dissection damage (Lu et al 2006) and that such dissection damage can release growth factors such as FGF-2 (Vincent et al 2002). In this study it may be that release of endogenous FGF-2 is stimulating the chondrocytes to respond and that the dissection of the cartilage out of the joint and its handling is sufficient to initiate the response.

The experiments described in this paper clearly demonstrate that chondrocytes have the ability to leave the parent cartilage and migrate into an adjacent gelatin matrix following single impact load damage. This process of migration might be expected to be associated with an alteration in cell shape i.e. the cell might form a more flattened, fibroblastic shape in order to move through the matrix. Our studies revealed no consistent increase in elongated cells across the experiments where the products of migration were detected, indicating that gross shape change to an elongated phenotype does not appear to be an indicator of motility in this model. Cell motility is a complex phenomenon in which the cytoskeleton (predominately actin and vimentin) plays an essential role [24] and more detailed observations of the microstructure of the chondrocytes in this experimental model is warranted.

The addition of 100 ng/ml BMP-2 to the experimental model significantly decreased the response of the chondrocytes – the numbers of cells within the gelfoam, extruding and on the articular surface were reduced. The mechanism for this is not known, however, there are many different ways in which BMP-2 could inhibit these responses. It is known that factors involved in the extrusion and migration process of chondrocytes are the upregulation/activation of catabolic enzymes such as matrix metalloproteinases (MMPs) that degrade the matrix permitting the movement of chondrocytes through. BMP-2 has been shown to have an inhibitory effect on the expression and activity of various MMPs in a number of experimental situations [25,26] and it is possible that the inhibitory action of BMP-2 may occur via this route.

In our previous work we have demonstrated that FGF-2 can induce a repair response and other workers have demonstrated the release of FGF-2 by dissection and load (Lu et al 2006, Vincent et al 2004, 2005). If FGF-2 is driving the response of the cells out of the cartilage then it may be that BMP-2 is, in some way, inhibiting at the level of FGF-2. The individual roles played by BMP-2 and FGF-2 differ in any given tissue and experimental system. However, in some systems BMP-2 and FGF-2 are an antagonistic pair [27]. Experiments in transgenic mice has demonstrated that BMPs antagonize FGF signalling by inhibiting at least two of the intracellular pathways activated by FGFs, namely STAT and ERK. One possible way in which BMPs may inhibit STAT and ERK1/2 is by negatively regulating the expression of FGF signalling components [27]. Studies have shown that BMP and FGF signalling have opposing actions in the growth [28], however, limb-culture studies have yielded contradictory results; some studies suggest that BMPs exert stimulatory effects on differentiation, whilst others provide evidence to support an inhibitory effect.

In conclusion this study has shown that cartilage subjected to damage and culture has the ability to respond to damage by activating chondrocytes to migrate to areas of damage and to migrate out of the parent cartilage into a 3D gelatin scaffold. These results agree well with those of Lu *et al *(2006) who demonstrated that mechanical fragmentation of cartilage mobilized chondrocytes to migrate and redistribute into a scaffold. These authors suggest the delivery of chondrocytes in the form of cartilage tissue fragments in conjunction with appropriate polymeric scaffolds could provide a novel intraoperative approach for cell-based cartilage repair. The results presented in this study agree with these authors' observations. Taken together these two papers indicate that, under the appropriate circumstances, chondrocytes can respond and migrate to areas of damage where they remain phenotypically stable and can aid in the repair of cartilage lesions. The question then arises – why does this not happen *in vivo *in naturally occurring disease. It can be hypothesized that BMP-2, demonstrated here to inhibit the damage/repair response, is inhibiting the response i*n vivo*, blocking the joint's ability to repair itself and clearly warrants further investigation.

## Conclusion

These studies demonstrate that articular chondrocytes can be stimulated to migrate out of parent cartilage following single impact load and culture. The addition of BMP-2 to the culture medium quantitatively reduced the repair response. It may be that the inhibitory effect of BMP-2 in this experimental model provides a clue to the apparent inability of articular cartilage to heal itself following damage *in vivo*.

## Competing interests

The author(s) declare that they have no competing interests.

## Authors' contributions

FH conceived the study and designed the study. TV carried out the practical experimental work. FH drafted the manuscript. Both authors read and approved the final manuscript.

## Pre-publication history

The pre-publication history for this paper can be accessed here:



## References

[B1] Hunziker EB, Rosenberg LC (1996). Repair of partial-thickness defects in articular cartilage: cell recruitment from the synovial membrane. J Bone Joint Surg Am.

[B2] Buckwalter JA, Mankin HJ (1998). Articular cartilage: degeneration and osteoarthritis, repair, regeneration, and transplantation. Instr Course Lect.

[B3] Henson FMD, Davies ME (2005). Promotion of the intrinsic damage-repair response in articular cartilage by FGF-2. Osteoarthritis and Cartilage.

[B4] Lu Y, Dhanaraj S, Wang Z, Bradley DM, Bowman SM, Cole BJ, Binette F (2006). Minced cartilage without cell culture serves as an effective intraoperative cell source for cartilage repair.. Journal of Orthpaedic Researchj.

[B5] Vincent T, Hermansson MA, Bolton M, Wait R, Saklatvala J (2002). Basic FGF mediates an immediate response of articular cartilage to mechanical injury. Proceedings of the National Academy of Sciences USA.

[B6] Vincent T, Hermansson MA, Hansen UN, Amis AA, Saklatvala J (2004). Basic fibroblastic growth factor mediates transduction of mechanical signals when articular cartilage is loaded.. Arthritis and Rheumatism.

[B7] Jin EJ, Lee SY, Choi YA, Jung JC, Bang OS, Kang SS (2006). BMP-2-enhanced chondrogenesis involves p38 MAPK-mediated down-regulation of Wnt-7a pathway. Mol Cells.

[B8] Wei Y, Hu Y, Lv R, Li D (2006). Regulation of adipose-derived adult stem cells differentiating into chondrocytes with the use of rhBMP-2.. Cytotherapy.

[B9] Dell'Accio F, De Bari C, El Tawil NM, Barone F, Mitsiadis TA, O'Dowd J, Pitzalis C (2006). Activation of WNT and BMP signaling in adult human articular cartilage following mechanical injury. Arthritis Research and Therapeutics.

[B10] Suzuki T, Bessho K, Fujimura K, Okubo Y, Segami N, Iizuka T (2002). Regeneration of defects in the articular cartilage in rabbit temporomandibular joints by bone morphogenetic protein-2. British Journal of Oral and Maxillofacial Surgery.

[B11] Bowe EA, Henson FMD, Caddick J, Jeffcott LB, Davies ME (2004). Response of equine cartilage to single impact load. Online Journal of Veterinary Research.

[B12] Jeffrey JE, Gregory DW, Aspden RM (1995). Matrix damage and chondrocyte viability following a single impact load on articular cartilage. Arch Biochem Biophys.

[B13] Hicks DL, Sage AB, Shelton E, Schumacher BL, Sah RL, Watson D (2007). Effect of BMPs 2 and 7 on septal chonrdocytes in alginate. Otolaryngeal Head and Neck Surgery.

[B14] Nöth U, Rackwitz L, Heymer A, Weber M, Baumann B, Steinert A, Schütze N, Jakob F, Eulert J (2007). Chondrogenic differentiation of human mesenchymal stem cells in collagen type I hydrogels. J Biomed Mater Res A.

[B15] Henson FMD, Davies ME, Schofield PN, Jeffcott LB (1996). Expression of types II, VI and X collagen in equine growth cartilage during development.. Equine Veterinary Journal.

[B16] Murray RC, Janicke HC, Henson FM, Goodship A (2000). Equine carpal articular cartilage fibronectin distribution associated with training, joint location and cartilage deterioration. Equine Vet J.

[B17] Henson FMD, Bowe EA, Davies ME (2005). Promotion of the intrinsic damage-repair response in articular cartilage by fibroblastic growth factor-2. Osteoarthritis Cartilage.

[B18] Huser CA, Davies ME (2006). Validation of an in vitro single-impact load model of the initiation of osteoarthritis-like changes in articular cartilage. Journal of Orthopaedic Research.

[B19] Baek CH, Lee JC, Jung YG, Ko YJ, Yoon JJ, Park TG (2002). Tissue-engineered cartilage on biodegradable macroporous scaffolds: cell shape and phenotypic expression.. Laryngoscope.

[B20] Gugala Z, Gogolewsk S (2000). In vitro growth and activity of primary chondrocytes on a resorbable polylactide three-dimensional scaffold.. Journal of Biomedical Materials Research.

[B21] Itoh N, Mima T, Mikawa T (1996). Loss of fibroblast growth factor receptors is necessary for terminal differentiation of embryonic limb muscle. Development.

[B22] Montell DJ (1999). The genetics of cell migration in Drosophila melanogaster and Caenorhabditis elegans developmen. Development.

[B23] Rickard A, Portell C, Siegal J, Goeckeler Z, D L (2003). Measurement of the motility of endothelial cells in confluent monolayers. Microcirculation.

[B24] Maree AF, Jilkine A, Dawes A, Grieneisen VA, Edelstein-Keshet L (2006). Polarization and movement of keratocytes: a multiscale modelling approach.. Bulletin of Mathematical Biology.

[B25] Kumagai T, Shimizu T, Takeda K (2006). Bone morphogenetic protein-2 suppresses invasiveness of TSU-Pr1 cells with the inhibition of MMP-9 secretion. Anticancer Res.

[B26] Takiguchi T, Kobayashi M, Suzuki R, Yamaguchi A, Isatsu K, Nishihara T, Nagumo M, Hasegawa K (1998). Recombinant human bone morphogenetic protein-2 stimulates osteoblast differentiation and suppresses matrix metalloproteinase-1 production in human bone cells isolated from mandibulae. Journal of Periodontal Research.

[B27] Yoon BS, Pogue R, Ovchinnikov DA, Yoshii I, Mishina Y, Behringer RR, Lyons KM (2006). BMPs regulate multiple aspects of growth-plate chondrogenesis through opposing actions on FGF pathways. Development.

[B28] Minina E, Kreschel C, Naski MC, Ornitz DM, Vortkamp A (2002). Interaction of FGF, Ihh/Pthlh, and BMP signalling integrates chondrocyte proliferation and hypertrophic differentiation. Development and Cell.

